# Supplementary material for: Reduced temporal and spatial stability of neural activity patterns predict cognitive control deficits in children with ADHD
Source: Nat Commun. 2025 Mar 8;16:2346. doi: 10.1038/s41467-025-57685-x (PMC11890578; doi:10.1038/s41467-025-57685-x)
Supplement: Supplementary file 1 — Supplementary Information [file 41467_2025_57685_MOESM1_ESM.pdf]

Supplementary Materials

Reduced temporal and spatial stability of neural activity patterns predict cognitive control deficits in children with ADHD

Zhiyao Gao[1], Katherine Duberg[1], Stacie L Warren[2], Li Zheng[3], Stephen P. Hinshaw[4,5], Vinod Menon[1,6,7,8], Weidong Cai[1,6,8]

Supplementary Methods

Normality of distribution of trial-evoked neural responses

Prior to assessing the temporal variability of trial-evoked neural responses, we tested determine whether it is appropriate to fit trial-evoked neural responses in the Gaussian model. The test specifically targeted 17 ROIs of the inhibitory control system, as identified by an independent meta-analysis study [1]. The β time series for each trial type (e.g., Uncertain Go, Certain Go, and Stop) were extracted from the peak voxel within each ROI for each participant, and subsequently fitted with a normal distribution. The assessment of normality was performed using the 'Chi2gof' function available in Matlab 2020. The proportion of non-normally distributed data was computed for each ROI and condition.

Consistency of recruitment within the salience and frontoparietal network between proactive and reactive control

Our study found that both proactive and reactive control engaged similar brain networks, particularly within the salience and frontoparietal regions. However, there are notable differences in the consistency of recruitment within these networks between typically developing (TD) children and children with ADHD. To validate this observation, we investigated the spatial consistency of recruitment of proactive and reactive control within the salience and frontoparietal network for each group. Reactive control was identified using the conventional univariate contrast between Successful Stop trials and Uncertain Go trials, while proactive control was delineated by the contrast between Uncertain Go and Certain Go trials. *Pearson's* correlation was used to assess the consistency of recruitment between these two control processes by correlating the statistic maps within the salience and frontoparietal network.

Controlling the potential confounding factors of age, gender and head motion

To ensure the robustness of our findings against potential confounding factors, we conducted supplementary ROI-based analyses. Specifically, we performed linear regression analyses with group (TD coded as 0, ADHD as 1) as the independent variable, while controlling for age, gender, and head motion as covariates of no interest. The dependent variables in these analyses were brain measures, including temporal and spatial stability. Cook's distance was employed to account for potential outliers and mitigate their influence.

Supplementary Results

Power analysis

To determine the appropriate sample size for detecting the expected effect size, we conducted a power analysis based on data from our previous study [2]. That study employed a similar go/no-go task focusing on inhibitory control in children with and without ADHD, within a comparable age range. The analysis indicated that a total sample of 60 participants would provide 80% power with an alpha level of 0.05.

Normality of distribution of trial-evoked neural responses

We found that trial-evoked neural responses fit a normal distribution for most of our participants, ranging between 77% and 97% (see **Supplementary Table S2** for overall ratio). There were no significant between-group differences in non-normality (all *p*s > 0.2, see **Table S3-S4** for TD and ADHD separately).

Consistency of recruitment within the salience and frontoparietal network between proactive and reactive control

In comparison to children with ADHD (r=0.05), TD children (r=0.17) exhibited significantly higher consistency in recruitment of the SN and FPN between proactive and reactive control (z=14.94, p<0.001).

Robustness with respect to age, gender, and head motion

To assess the robustness of our findings against potential confounding factors, including age, gender, and head motion, we conducted additional ROI-based analyses for between-group comparisons while controlling for these variables. We selected ROIs implicated in cognitive control from salience (SN), encompassing the right anterior insula (rAI) and presupplementary motor cortex (preSMA), and frontal-parietal regions (FPN), encompassing right dorsal lateral prefrontal cortex (rdlPFC), and right posterior parietal cortex (rPPC) (see Methods and Figure 1h).

Increased temporal variability of trial-evoked brain responses in children with ADHD after accounting for age, gender, and head motion

In comparison to TD children, children with ADHD exhibited significantly higher standard deviation in the preSMA, rDLPFC, and rPPC during Uncertain and Certain Go trials, in all four ROIs during Stop trials after accounting for age, gender, and head motion (all ps<0.002, FDR corrected). Children only showed lower standard deviation in the rAI during Uncertain and Certain Go trials (*p*s < 0.001, FDR corrected), see **Table S5**. Moreover, children with ADHD had significantly higher kurtosis than TD children in the rAI during Uncertain Go trials, and the rPPC during Certain Go trials (all *p*s<0.006, FDR corrected) after controlling for age, gender, and head motion. FDR correction was applied to four ROIs, see **Table S6**.

Weaker spatial stability of trial-evoked brain responses in children with ADHD after accounting for age, gender, and head motion

In comparison to TD children, children with ADHD exhibited significant lower spatial stability in the preSMA and rPPC across all three trial types (ps < 0.05, FDR corrected) after controlling the effect of age, gender, and head motion, see **Table S7**.

Weakened association between proactive control system and behavioral regulation in ADHD after accounting for age, gender, and head motion

In comparison to TD children, children with ADHD exhibited a significantly weaker association between the inhibitory control pattern (ICP) and reaction time in the preSMA, rAI, and rDLPFC during Uncertain Go trials after accounting for age, gender, and head motion (ps $\leqslant 0.015$, FDR corrected). This weaker association was also observed in the preSMA when both Uncertain and Certain Go trials were combined ($p = 0.004$, FDR corrected), after controlling for age, gender, and head motion, see **Table S8**.

Supplementary Figures

Figure S1. Children with ADHD showed lower skewness of trial-evoked brain responses in comparison to TD children (*p*<0.05, corrected).

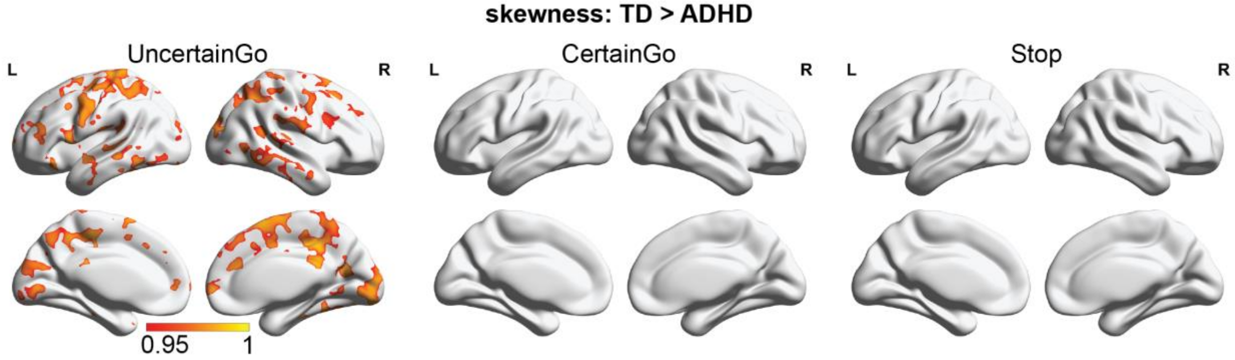

Figure S2. Children with ADHD showed significantly higher standard deviations of neural activity compared to TD children across all three trial types. Children with ADHD also exhibited significantly greater kurtosis during Uncertain and Certain Go trials compared to TD children. TD children demonstrated greater skewness of neural activity during Uncertain Go trials when compared to children with ADHD. All results underwent TFCE correction, *p*<0.05 (of note, the significance map represents values of 1-p). Analyses were conducted with additional head motion control.

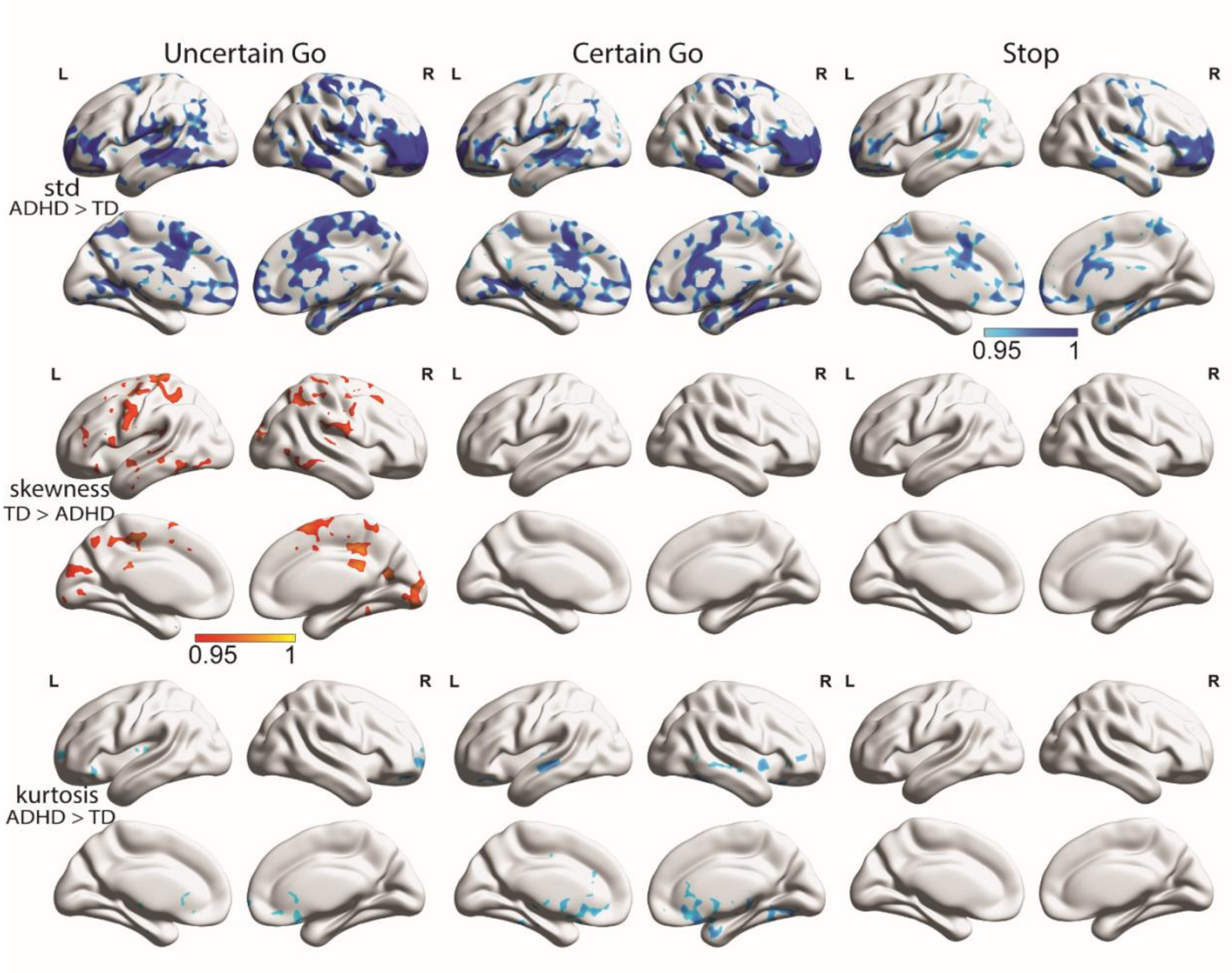

Figure S3. In comparison to children with ADHD, TD children demonstrated significant greater spatial stability of neural activity patterns across three trial types after controlling head motion (TFCE, $p<0.05$, the significance map represents values of 1 minus p).

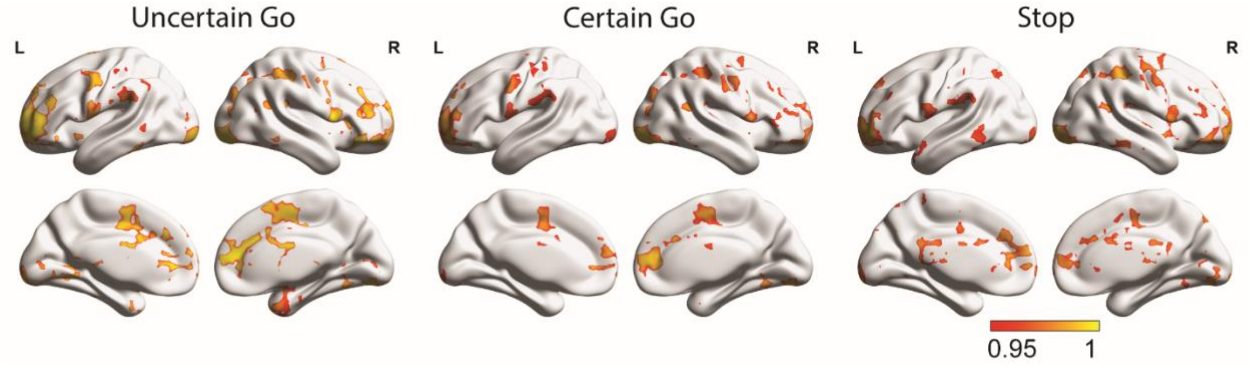

Figure S4. Weak association between trial-evoked inhibition-alike brain response and RT fluctuation in children with ADHD during Go trials (TFCE, $p<0.05$, the significance map represents values of 1 minus p). Analyses were conducted with additional head motion control.

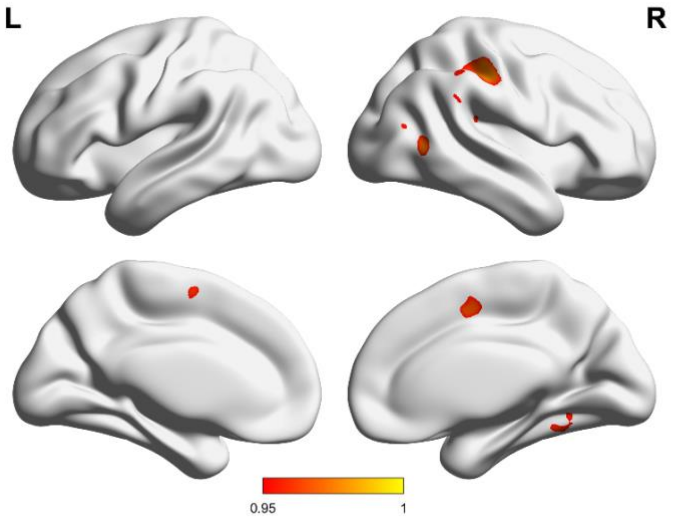

Supplementary Tables

Table S1. Demographic and behavioral statistics of behavioral-only sample, including all participants who meet the behavioral criteria regardless of their head motion.

Demographics	TD Controls (N=37)	ADHD (N = 50)	p-value
Age (SD)	10.46 (1.07)	10.66 (1.20)	0.406
Gender (Male/Female)	23/14	35/15	0.443
IQ (SD)	113.649 (13.292)	102.75 (14.179)	p<0.001
Inattention score	49.55	79.85	p<0.001
Hyperactivity score	49.32	79.09	p<0.001
**Cued SST Performance**			
Certain Go Accuracy (%)	92.5	90.8	0.234
Uncertain Go Accuracy (%)	94.8	91.4	0.017
Stop Accuracy (%)	52.2	52.4	0.898
SSRT (ms)	296.6	337.4	0.016
RT slowing (ms)	23.7	25.1	0.881
Certain Go RT (ms)	483.4	534.5	0.004
Uncertain Go RT (ms)	507.1	559.6	0.002
**ex-Gaussian modelling**			
Certain Go RT (mu)	395.7	423.3	0.009
Certain Go RT (sigma)	109.8	134.9	0.206
Certain Go RT (tau)	87.7	112.2	0.021
Uncertain Go RT (mu)	418.4	438.5	0.055
Uncertain Go RT (sigma)	50.2	50.0	0.968
Uncertain Go RT (tau)	88.7	121.1	0.002

A single-tailed chi-square test was performed to assess the presence of gender differences between children with ADHD and TD children. For all other between-group comparisons, two tailed unpaired t-tests were utilized. TD: typically developing; ADHD: attention deficit/hyperactivity disorder.

Table S2. The percentage of participants whose trial-evoked neural responses rejected the null hypothesis of normal distribution.

ROI name	Uncertain Go	Certain Go	Stop
rAI	0.15	0.1	0.11
rSFG	0.11	0.05	0.11
rFP	0.07	0.13	0.05
rSPL	0.07	0.11	0.05
rSMG	0.1	0.1	0.03
lAI	0.1	0.18	0.08
rSTG	0.07	0.15	0.05
rThalamus	0.17	0.15	0.13
lSMG (posterior)	0.15	0.1	0.07
rMFG	0.07	0.08	0.1
lSMG (anterior)	0.07	0.11	0.07
Precuneus	0.05	0.07	0.07
lSPL	0.05	0.08	0.11
lSFG	0.07	0.07	0.11
lMFG	0.13	0.23	0.1
lOG	0.15	0.11	0.1
rSFG/Precentral	0.13	0.1	0.05
Averaged	0.1	0.11	0.08

Sample size, N = 61. rAI, right anterior insula; rSFG, right superior frontal gyrus; rFP, right frontal pole; rSPL, right superior parietal lobe; rSMG, right supramarginal gyrus; lAI, left anterior insula; rSTG, right superior temporal gyrus; rThalamus, right thalamus; lSMG, left supramarginal gyrus; lSPL, left superior parietal lobe; lSFG, left superior frontal gyrus; lOG, inferior occipital gyrus.

Table S3. The percentage of participants whose trial-evoked neural responses rejected the null hypothesis of normal distribution (TD).

ROI name	Uncertain Go	Certain Go	Stop
rAI	0.11	0.09	0.17
rSFG	0.14	0.09	0.17
rFP	0	0.11	0.06
rSPL	0.03	0.09	0.03
rSMG	0.14	0.09	0.03
lAI	0.06	0.11	0.06
rSTG	0.09	0.14	0.06
rThalamus	0.18	0.18	0.12
lSMG (posterior)	0.17	0.06	0.06
rMFG	0.03	0.09	0.09
lSMG (anterior)	0.03	0.03	0.06
Precuneus	0	0.09	0.06
lSPL	0	0.06	0.14
lSFG	0.09	0.06	0.14
lMFG	0.11	0.29	0.09
lOG	0.11	0.14	0.11
rSFG/Precentral	0.11	0.09	0.09
Averaged	0.08	0.10	0.09

Sample size, N = 35. rAI, right anterior insula; rSFG, right superior frontal gyrus; rFP, right frontal pole; rSPL, right superior parietal lobe; rSMG, right supramarginal gyrus; lAI, left anterior insula; rSTG, right superior temporal gyrus; rThalamus, right thalamus; lSMG, left supramarginal gyrus; lSPL, left superior parietal lobe; lSFG, left superior frontal gyrus; lOG, inferior occipital gyrus

Table S4. The percentage of participants whose trial-evoked neural responses rejected the null hypothesis of normal distribution (ADHD).

ROI name	Uncertain Go	Certain Go	Stop
rAI	0.19	0.12	0.04
rSFG	0.08	0	0.04
rFP	0.15	0.15	0.04
rSPL	0.12	0.15	0.08
rSMG	0.04	0.12	0.04
lAI	0.15	0.27	0.12
rSTG	0.04	0.15	0.04
rThalamus	0.15	0.12	0.15
lSMG (posterior)	0.11	0.15	0.08
rMFG	0.12	0.08	0.12
lSMG (anterior)	0.12	0.23	0.08
Precuneus	0.12	0.04	0.08
lSPL	0.12	0.12	0.08
lSFG	0.04	0.08	0.08
lMFG	0.15	0.15	0.12
lOG	0.19	0.08	0.08
rSFG/Precentral	0.15	0.12	0
Averaged	0.12	0.12	0.07

Sample size, N = 26. rAI, right anterior insula; rSFG, right superior frontal gyrus; rFP, right frontal pole; rSPL, right superior parietal lobe; rSMG, right supramarginal gyrus; lAI, left anterior insula; rSTG, right superior temporal gyrus; rThalamus, right thalamus; lSMG, left supramarginal gyrus; lSPL, left superior parietal lobe; lSFG, left superior frontal gyrus; lOG, inferior occipital gyrus

Table S5. Increased temporal variability (standard deviation) of trial-evoked brain responses in children with ADHD (FDR corrected).

Brain measures	Conditions	Slope	p-value
Uncertain Go	preSMA	0.248	p < 0.001
	rAI	-0.390	p < 0.001
	rdlPFC	0.601	p = 0.001
	rPPC	0.373	p < 0.001
Certain Go	preSMA	0.214	p < 0.001
	rAI	-0.382	p < 0.001
	rdlPFC	0.579	p < 0.001
	rPPC	0.434	p < 0.001
Stop	preSMA	0.104	p < 0.001
	rAI	0.121	p < 0.001
	rdlPFC	0.127	p < 0.001
	rPPC	0.086	p < 0.002

Two tailed linear regression analysis were conducted, the slopes were compared against zero. Sample size, 35 TD children and 26 ADHD children. rAI, right anterior insula; preSMA, presupplementary motor cortex; rdlPFC, right dorsal lateral prefrontal cortex; rPPC, right posterior parietal cortex.

Table S6. Increased temporal variability (kurtosis) of trial-evoked brain responses in children with ADHD (FDR corrected).

Brain measures	Conditions	Slope	p-value
Uncertain Go	preSMA	0.210	0.445
	rAI	0.408	0.026
	rdlPFC	0.104	0.445
	rPPC	0.188	0.445
Certain Go	preSMA	0.077	0.402
	rAI	0.180	0.402
	rdlPFC	0.077	0.402
	rPPC	0.318	0.015
Stop	preSMA	0.070	0.708
	rAI	0.134	0.708
	rdlPFC	0.243	0.558
	rPPC	0.141	0.374

Two tailed linear regression analysis were conducted, the slopes were compared against zero. Sample size, 35 TD children and 26 ADHD children. rAI, right anterior insula; preSMA, presupplementary motor cortex; rdlPFC, right dorsal lateral prefrontal cortex; rPPC, right posterior parietal cortex.

Table S7. Weakened spatial stability of trial-evoked brain responses in children with ADHD (FDR corrected).

Brain measures	Conditions	Slope	p-value
Uncertain Go	preSMA	-0.034	0.004
	rAI	-0.025	0.119
	rdlPFC	-0.032	0.119
	rPPC	-0.028	0.011
Certain Go	preSMA	-0.011	0.039
	rAI	0.008	0.695
	rdlPFC	-0.032	0.108
	rPPC	-0.038	0.010
Stop	preSMA	-0.024	0.023
	rAI	-0.005	0.061
	rdlPFC	-0.045	0.339
	rPPC	-0.061	0.023

Two tailed linear regression analysis were conducted, the slopes were compared against zero. Sample size, 35 TD children and 26 ADHD children. rAI, right anterior insula; preSMA, presupplementary motor cortex; rdlPFC, right dorsal lateral prefrontal cortex; rPPC, right posterior parietal cortex.

Table S8. Weakened association between proactive control system and behavioral regulation in ADHD (FDR corrected).

Brain measures	Conditions	Slope	p-value
Uncertain Go	preSMA	-0.069	0.009
	rAI	-0.042	0.015
	rdlPFC	0.027	0.004
	rPPC	-0.053	0.134
Certain Go	preSMA	-0.014	0.121
	rAI	-0.066	0.121
	rdlPFC	-0.040	0.121
	rPPC	-0.023	0.818
Uncertain + Certain Go	preSMA	-0.050	0.004
	rAI	-0.048	0.054
	rdlPFC	0.008	0.054
	rPPC	-0.029	0.184

Two tailed linear regression analysis were conducted, the slopes were compared against zero. Sample size, 35 TD children and 26 ADHD children. rAI, right anterior insula; preSMA, presupplementary motor cortex; rdlPFC, right dorsal lateral prefrontal cortex; rPPC, right posterior parietal cortex.

Table S9. Averaged number of trials/pairs included in fMRI analysis by condition and group

	TD				ADHD			
	Uncertain Go	Certain Go	SuccStop	Stop	Uncertain Go	Certain Go	SuccStop	Stop
Trial Number	59.714	58.2	16.486	31.514	56.423	56.192	16.385	29.846
Pair Number	893.143	849.4	68.571	248.257	800.346	793.692	67.692	223.385

Sample size, N(TD) = 35, N(ADHD) = 26. SuccStop: Successful stop trials.

Table S10. Performance difference across sessions in TD children.

Index	Run1	Run2	p-value
Certain Go Accuracy (%)	91.8	93.3	0.198
Uncertain Go Accuracy (%)	94.1	94.8	0.948
Stop Accuracy (%)	51.8	53.0	0.361
SSRT (ms)	285.0	289.6	0.441
RT slowing (ms)	21.8	18.8	0.671
Certain Go RT (ms)	479.3	487.8	0.241
Uncertain Go RT (ms)	501.1	506.5	0.466
**ex-Gaussian modelling**			
Certain Go RT (mu)	397.6	407.6	0.167
Certain Go RT (sigma)	47.2	47.9	0.903
Certain Go RT (tau)	81.6	80.2	0.856
Uncertain Go RT (mu)	419.7	425.9	0.365
Uncertain Go RT (sigma)	41.8	45.0	0.476
Uncertain Go RT (tau)	81.5	80.7	0.929

One sample t-tests were utilized. N = 35. TD: typically developing.

Table S11. Performance difference across sessions in children with ADHD.

Index	Run1	Run2	p-value
Certain Go Accuracy (%)	92.9	92.8	0.946
Uncertain Go Accuracy (%)	93.3	93.4	0.908
Stop Accuracy (%)	53.6	54.3	0.523
SSRT (ms)	303.2	308.4	0.724
RT slowing (ms)	22.6	24.9	0.835
Certain Go RT (ms)	510.5	520.6	0.252
Uncertain Go RT (ms)	533.1	545.4	0.126
**ex-Gaussian modelling**			
Certain Go RT (mu)	428.7	416.4	0.125
Certain Go RT (sigma)	48.8	41.2	0.273
Certain Go RT (tau)	81.8	104.1	0.060
Uncertain Go RT (mu)	425.0	443.0	0.065
Uncertain Go RT (sigma)	42.8	41.0	0.839
Uncertain Go RT (tau)	107.9	102.4	0.626

One sample t-tests were utilized. N = 26. ADHD: attention deficit/hyperactivity disorder.

Table S12. Brain regions showing increased temporal variability of trial-evoked brain responses in children with ADHD – standard deviation

Index	Voxels	Max(t)	X	Y	Z	Brain Areas
Uncertain Go	40719	5.5500	16	-12	20	preSMA/anterior cingulate gyrus, bilateral frontal cortex, bilateral temporal and visual cortex, lateral and medial parietal cortex
	158	3.9700	-32	-58	50	Left superior parietal lobe
	36	5.2000	-18	46	28	Left frontal pole
	19	3.5900	4	36	32	Paracingulate gyrus
Certain Go	36038	5.6400	24	-34	58	Postcentral gyrus, preSMA/anterior cingulate gyrus, medial parietal cortex
	81	3.4800	24	-48	58	Right superior parietal lobule
	21	3.8700	48	16	42	Right middle frontal gyrus
	16	3.7000	-16	-14	-30	Parahippocampus
	16	4.1700	10	-80	52	Right occipital cortex
Stop	14056	5.6200	64	0	28	Right precentral gyrus, inferior frontal gyrus, lateral and medial temporal lobe
	99	3.4300	2	-2	48	preSMA
	34	4.3600	-10	-50	52	Left precuneus
	32	3.8100	8	-46	58	Right precuneus

Table S13. Brain regions showing increased temporal variability of trial-evoked brain responses in children with ADHD – kurtosis

Index	Voxels	Max(t)	X	Y	Z	Brain Areas
Uncertain Go	5107	4.8200	-22	60	6	Bilateral frontal pole/inferior frontal gyrus
	1682	4.1600	-30	-24	4	Left putamen/ventral medial PFC, medial temporal lobe
	616	4.6900	48	-38	60	Right supramarginal gyrus/postcentral gyrus
	326	4.6900	12	-4	62	Superior frontal gyrus/preSMA
	204	3.9200	52	-40	26	Right supramarginal gyrus
	145	4.3400	18	-48	54	Right superior parietal lobe
	79	3.8600	36	-24	2	Right posterior insula/planum polare
	67	4.0200	-56	-24	-10	Left middle temporal gyrus
	63	4.0800	-32	-50	58	Left superior parietal lobe
	33	4.0900	-28	-40	38	Left superior parietal lobe
	31	3.3300	-22	-24	8	Left thalamus
	28	3.5800	-34	30	48	Left middle frontal gyrus
	28	3.1400	56	10	24	Right precentral gyrus
	27	4.3500	28	-12	0	Right putamen/pallidum
	26	4.2300	-32	-46	70	Left superior parietal lobe
	26	3.5800	-56	-10	-12	Left superior temporal gyrus
	19	3.8200	-2	-22	-32	Brain stem
	14	3.8700	54	-50	8	Right middle temporal lobe
Certain Go	28673	4.7600	2	50	-10	Bilateral anterior insular, inferior frontal gyrus, superior frontal gyrus, preSMA, ventral medial PFC, lateral and medial temporal lobe; right lateral and medial parietal cortex
	36	3.6100	-54	-36	38	Left supramarginal gyrus
	31	5.0400	32	-80	28	Right lateral occipital cortex

Table S14. Brain regions showing reduced spatial stability of trial-evoked brain responses in children with ADHD.

Index	Voxels	Max(t)	X	Y	Z	Brain Areas
Uncertain Go	41193	5.0900	-34	62	-4	Left frontal pole/inferior frontal gyrus, right inferior frontal gyrus, bilateral lateral parietal cortex, preSMA, anterior cingulate gyrus
	283	3.6600	64	-50	-16	Right inferior temporal gyrus/medial temporal lobe
Certain Go	25456	4.5000	32	-96	-12	Right occipital pole, right lateral parietal cortex/postcentral gyrus, right inferior frontal gyrus, preSMA/anterior cingulate gyrus, posterior cingulate cortex
	603	4.4900	-30	-30	22	Left parietal operculum cortex
	470	3.5000	-64	-38	30	Left supramarginal gyrus
	426	3.7800	-16	-4	4	Left pallidum
	302	4.1300	-52	0	36	Left precentral gyrus /Left middle frontal gyrus
	168	3.6500	-36	10	20	Left precentral gyrus
Stop	18142	4.4800	-14	30	14	preSMA, cingulate gyrus, right inferior frontal gyrus, right anterior insular, right lateral parietal cortex, right postcentral gyrus
	7876	4.9400	-2	-74	-48	Cerebellum
	199	3.8500	16	-74	48	Lateral occipital cortex
	136	3.4800	46	-28	-18	Right inferior temporal lobe
	43	3.1500	58	-56	-18	Right inferior temporal lobe
	34	3.4300	36	4	-40	Right anterior temporal pole/temporal fusiform cortex
	17	3.5700	-62	8	22	Left precentral gyrus

Table S15. Brain regions showing spatial stability of proactive and reactive control in TD children.

Index	Voxels	Max(t)	X	Y	Z	Brain Areas
Proactive control	8926	5.3000	22	-56	-4	Lingual gyrus/medial visual cortex, occipital pole
	416	4.6800	32	20	8	Right anterior insular
	27	4.4900	36	40	14	Right frontal pole
	17	3.9000	54	12	22	Right inferior frontal gyrus
	16	3.9100	54	12	36	Right precentral gyrus
Reactive control	38016	6.8200	42	-66	-4	Bilateral visual, temporal, and parietal cortex, bilateral medial temporal lobe, bilateral anterior insular, inferior frontal gyrus, right superior frontal gyrus, posterior cingulate cortex, precuneus
	1936	5.6700	-24	18	14	PreSMA/anterior cingulate gyrus

Table S16. Brain regions showing spatial stability of proactive and reactive control in children with ADHD.

Index	Voxels	Max(t)	X	Y	Z	Brain Areas
Proactive control	343	5.7200	40	-70	-26	Right lateral occipital cortex
	116	5.8500	48	-76	4	Right lateral occipital cortex
	59	5.4700	-28	-72	-16	Cerebellum
Reactive control	3102	5.8200	42	-64	10	Right inferior occipital cortex
	1508	7.2500	-38	-72	8	Left inferior occipital cortex

Table S17. Brain regions showing association between inhibitory control pattern and trial-wise RT in TD children.

Index	Voxels	Max(t)	X	Y	Z	Brain Areas
Uncertain Go	22606	9.3300	-10	12	44	Paracingulate gyrus/superior frontal gyrus
	419	4.5400	-26	-60	0	Lingual gyrus
	54	4.9500	-32	-54	-38	Cerebellum
	34	3.4400	20	-56	34	Precuneus
	27	3.2900	12	-72	30	Cuneal cortex/precuneus
	20	4.4000	-36	-56	-10	Left temporal fusiform cortex
	20	3.4600	-6	-84	2	Intracalcarine cortex
	16	4.1400	-14	-46	10	Posterior cingulate gyrus
	22606	9.3300	-10	12	44	Paracingulate gyrus/superior frontal gyrus
Certain Go	32190	7.0700	14	16	38	Paracingulate gyrus/preSMA
	1588	5.3800	44	18	-8	Right anterior insular/frontal orbital cortex
	978	5.8600	4	-14	-16	Brain stem
	156	4.2700	34	44	34	Right frontal pole
	68	3.6800	14	10	-24	Frontal orbital cortex
	55	4.5500	-14	-32	6	Left thalamus
Uncertain Go & Certain Go	54154	9.3400	-10	10	42	Paracingulate gyrus/preSMA
	89	3.4200	26	-22	64	Right precentral gyrus
	58	2.9600	2	54	24	Paracingulate gyrus/superior frontal gyrus
	50	3.7100	-22	-38	-26	Left temporal fusiform
	16	2.5100	-2	-26	66	Precentral gyrus

Table S18. Brain regions showing association between inhibitory control pattern and trial-wise RT in children with ADHD.

Index	Voxels	Max(t)	X	Y	Z	Brain Areas
Uncertain Go	103	5.9900	-8	4	50	Supplementary motor cortex
Uncertain Go & Certain Go	1191	7.8500	6	10	46	Paracingulate gyrus/preSMA

Table S19. Brain regions showing stronger association between inhibitory control pattern and trial-wise RT in TD children compared to children with ADHD.

Index	Voxels	Max(t)	X	Y	Z	Brain Areas
Uncertain Go & Certain Go	6999	4.2200	52	-58	52	Right lateral occipital cortex/angular gyrus
	1269	4.7000	6	0	46	Paracingulate gyrus/preSMA
	1070	4.5100	44	16	-6	Left anterior insular
	330	4.4100	-34	-66	0	Left occipital fusiform gyrus
	284	3.9600	28	52	28	Right frontal pole
	98	3.6900	48	-2	48	Right precentral gyrus
	67	3.8400	30	0	-6	Right putamen
	30	3.6500	-20	-16	54	Left precentral gyrus
	26	3.5300	70	-26	-18	Right posterior middle temporal gyrus

Table S20. Brain regions showing greater inter-subject spatial stability of trial-evoked brain responses in TD children.

Index	Voxels	Max(t)	X	Y	Z	Brain Areas
Uncertain Go	32948	11.3000	-32	-96	-12	Left occipital pole, medial visual cortex
	3961	6.5300	42	52	24	Right frontal pole/inferior frontal gyrus
	710	6.6900	58	-50	50	Right angular gyrus/supramarginal gyrus
	172	4.3400	-10	-14	-34	Brain stem
	132	4.2700	64	-56	16	Right angular gyrus/lateral occipital cortex
	125	4.4600	-8	60	-20	Frontal pole
	106	4.6300	26	-60	26	Right precuneus/cuneal cortex
	53	7.8200	24	-88	-4	Right occipital fusiform gyrus
	47	5.6600	16	72	12	Right frontal pole
Certain Go	4134	12.8000	-42	-14	66	Left precentral/postcentral gyrus
	695	8.0700	-2	-62	-18	Cerebellum
	460	6.5300	-50	8	4	Left precentral gyrus
	433	5.8300	-22	0	-10	Left putamen
	292	7.8000	-42	56	10	Left frontal pole
	150	6.3600	-62	8	26	Left precentral gyrus
	135	5.1000	-8	0	66	PreSMA/superior frontal gyrus
	112	5.4200	0	-12	52	preSMA
	37	5	-6	-2	44	Anterior cingulate gyrus
	33	4.8200	-42	-10	-2	Left insular cortex
	22	5.3200	24	52	-10	Right frontal pole
Stop	276	5.2700	36	-50	38	Right angular gyrus
	107	4.7800	22	-68	58	Right lateral occipital cortex
	90	5.8100	-24	-54	-28	Cerebellum

Table S21. Brain regions showing greater inter-subject spatial stability of trial-evoked brain responses in children with ADHD.

Index	Voxels	Max(t)	X	Y	Z	Brain Areas
Uncertain Go	2456	10.3000	26	-100	6	Occipital pole
	955	7.4400	36	-66	34	Right lateral occipital cortex
	625	6.7200	-40	58	8	Left frontal pole
	590	6.0600	12	-62	24	Precuneus/suparcalcarine
	141	4.9900	26	60	8	Right frontal pole
	38	4.6000	-20	-50	42	Precuneus/posterior cingulate gyrus
	37	5.5300	-32	-58	48	Left superior parietal lobe
	34	5.5400	-28	-84	38	Left lateral occipital cortex
Certain Go	332	5.1000	34	-94	-14	Right occipital pole
	316	5.2500	-28	-98	-16	Left occipital pole
	288	5.4200	-32	-64	0	Left occipital fusiform gyrus
	81	5.2900	54	-58	50	Right lateral occipital cortex/angular gyrus
	57	4.6700	24	-76	42	Right lateral occipital cortex
	27	4.3300	10	-66	42	Right precuneus
	16	4.9200	18	-54	50	Right superior parietal lobe
Stop	9645	7.0800	-8	-22	12	Thalamus
	405	5.3000	42	40	-2	Right frontal pole
	268	5.9300	-52	-32	60	Left postcentral gyrus
	260	6.1800	48	-80	-18	Right lateral occipital cortex
	251	5.4700	-56	6	42	Left precentral gyrus/middle frontal gyrus
	56	5.2300	-14	62	-18	Left frontal pole
	18	5.1900	30	-12	66	Right precentral gyrus/superior frontal gyrus

Table S22. Brain regions showing greater inter-subject spatial stability during proactive and reactive control in TD children.

Index	Voxels	Max(t)	X	Y	Z	Brain Areas
Proactive control	51367	16.6000	36	-94	0	Occipital pole, medial and lateral visual and temporal areas, preSMA, bilateral inferior and middle frontal gyrus, precuneus
	108	8.8400	-32	64	10	Left frontal pole
Reactive control	66110	13.7000	22	-78	-8	Right occipital fusiform gyrus, bilateral visual and temporal regions, lateral and medial frontal cortex
	129	4.9700	8	-32	-50	Brain stem
	29	3.2400	16	54	-10	Right frontal pole

Table S23. Brain regions showing greater inter-subject spatial stability during proactive and reactive control in children with ADHD.

Index	Voxels	Max(t)	X	Y	Z	Brain Areas
Proactive control	6158	12.7000	-42	-86	-10	Left lateral occipital cortex
	3591	10.1000	44	-84	-14	Right lateral occipital cortex
	2107	9.4800	-44	-16	64	Left precentral gyrus
	598	5.2600	-54	-16	20	Left central opercular cortex/postcentral gyrus
	508	5.5600	28	-2	72	Right precentral gyrus
	350	5.9500	-22	2	-8	Left putamen
	247	6.2300	-50	10	6	Left inferior frontal gyrus
	216	7.3900	4	4	46	preSMA
	63	6.3500	32	-62	30	Right lateral occipital cortex
	13	5.3700	40	-2	0	Right insular
Reactive control	56325	10.8000	-34	-88	-18	Left lateral occipital cortex, medial visual temporal cortex, bilateral anterior insular, inferior frontal gyrus, medial frontal cortex
	106	4	-24	-46	74	Left superior parietal lobe
	67	4.7300	-44	-10	-18	Left planum polare
	14	4.9900	2	68	-8	Frontal pole

Table S24. Brain regions showing group-specific inter-subject spatial stability during proactive and reactive control in TD children.

Index	Voxels	Max(t)	X	Y	Z	Brain Areas
Proactive control	17358	10.8000	-28	-98	-12	Left occipital pole
	1479	6.0700	-30	-20	44	Precentral gyrus
	796	7.7700	52	-44	58	Right supramarginal gyrus
	651	4.9300	10	-54	54	Precuneus
	457	6.5400	52	12	-8	Right temporal pole
	381	5.8100	22	-84	40	Right lateral occipital cortex
	373	5.9000	-62	-52	2	Left middle temporal gyrus
	292	4.5300	64	0	-26	Right middle temporal gyrus
	204	5.0700	58	-30	28	Right parietal operculum cortex
	148	4.9200	-68	-22	30	Left supramarginal gyrus
	122	5.8000	36	64	2	Right frontal pole
	103	5.1700	54	-42	-2	Right middle temporal gyrus
	74	4.3500	-32	-76	48	Left occipital cortex
	54	3.3800	-52	-12	28	Left postcentral gyrus
	50	5.1800	-40	36	40	Left middle frontal gyrus/frontal pole
	35	4.4900	-42	8	42	Left middle frontal gyrus
	18	3.6600	54	-8	-44	Right inferior temporal gyrus
Reactive control	2846	7.8200	38	-34	50	Right postcentral gyrus
	2085	7.6400	28	-100	6	Right occipital pole
	899	6.9400	28	16	10	Right insular cortex
	542	7.4800	-28	-84	36	Left lateral occipital cortex
	342	8.9700	-30	-74	-10	Left occipital fusiform gyrus
	255	6.6300	-8	-50	12	Precuneus
	182	5.6900	-30	66	-2	Left frontal pole
	176	5.1000	40	-46	-6	Right temporal fusiform cortex
	90	4.9700	-14	-68	12	Intracalcarine cortex
	74	5.0900	26	54	16	Right frontal pole
	21	3.5000	40	24	50	Right middle frontal gyrus
	19	3.5000	-46	-32	-18	Left inferior temporal gyrus
	15	4.4100	30	46	30	Right frontal pole
	13	3.7200	48	-14	-14	Right middle temporal gyrus

Table S25. Brain regions showing group-specific inter-subject spatial stability during proactive and reactive control in children with ADHD.

Index	Voxels	Max(t)	X	Y	Z	Brain Areas
Proactive control	2925	8.8600	-36	-96	-8	Left occipital pole
	400	5.9800	42	-84	-18	Left lateral occipital cortex
	217	5.8800	56	-54	46	Right angular gyrus
	135	5.5900	42	4	38	Right precentral gyrus
	65	5.7100	36	-76	-8	Right lateral occipital cortex
	18	3.4300	0	-34	-2	Brain stem
	14	4.3800	26	-86	-2	Right occipital fusiform gyrus
Reactive control	1183	7.2900	28	-100	6	Right occipital pole
	614	5.4300	36	-66	34	Right lateral occipital cortex
	180	6.2000	20	4	4	Right putamen
	123	4.9100	40	4	-6	Right insular cortex
	99	5.9000	-32	-70	28	Left lateral occipital cortex
	88	6.6400	22	-46	46	Right superior parietal lobe
	63	4.3200	14	-78	24	Cuneal cortex
	16	7.3000	-12	10	48	PreSMA
	14	5.5800	-10	-90	2	Left occipital pole

Supplementary References

1 Cai, W. *et al.* Hyperdirect insula-basal-ganglia pathway and adult-like maturity of global brain responses predict inhibitory control in children. *Nature Communications* **10**, 4798 (2019). https://doi.org/10.1038/s41467-019-12756-8

2 Cai, W., Griffiths, K., Korgaonkar, M. S., Williams, L. M. & Menon, V. Inhibition-related modulation of salience and frontoparietal networks predicts cognitive control ability and inattention symptoms in children with ADHD. *Molecular psychiatry* **26**, 4016-4025 (2021).
