# Supplementary material for: Reduced temporal and spatial stability of neural activity patterns predict cognitive control deficits in children with ADHD
Source: Nat Commun. 2025 Mar 8;16:2346. doi: 10.1038/s41467-025-57685-x (PMC11890578; doi:10.1038/s41467-025-57685-x)
Supplement: Supplementary file 2 — Reporting Summary [file 41467_2025_57685_MOESM2_ESM.pdf]

nature portfolio

Corresponding author(s): Zhiyao Gao; Weidong Cai; Vinod Menon

Last updated by author(s): Jan 20, 2025

Reporting Summary

Nature Portfolio wishes to improve the reproducibility of the work that we publish. This form provides structure for consistency and transparency in reporting. For further information on Nature Portfolio policies, see our Editorial Policies and the Editorial Policy Checklist.

Statistics

For all statistical analyses, confirm that the following items are present in the figure legend, table legend, main text, or Methods section.

n/a	Confirmed	
☐	☒	The exact sample size (*n*) for each experimental group/condition, given as a discrete number and unit of measurement
☐	☒	A statement on whether measurements were taken from distinct samples or whether the same sample was measured repeatedly
☐	☒	The statistical test(s) used AND whether they are one- or two-sided<br>*Only common tests should be described solely by name; describe more complex techniques in the Methods section.*
☐	☒	A description of all covariates tested
☐	☒	A description of any assumptions or corrections, such as tests of normality and adjustment for multiple comparisons
☐	☒	A full description of the statistical parameters including central tendency (e.g. means) or other basic estimates (e.g. regression coefficient) AND variation (e.g. standard deviation) or associated estimates of uncertainty (e.g. confidence intervals)
☐	☒	For null hypothesis testing, the test statistic (e.g. *F*, *t*, *r*) with confidence intervals, effect sizes, degrees of freedom and *P* value noted<br>*Give P values as exact values whenever suitable.*
☒	☐	For Bayesian analysis, information on the choice of priors and Markov chain Monte Carlo settings
☒	☐	For hierarchical and complex designs, identification of the appropriate level for tests and full reporting of outcomes
☐	☒	Estimates of effect sizes (e.g. Cohen's *d*, Pearson's *r*), indicating how they were calculated

Our web collection on statistics for biologists contains articles on many of the points above.

Software and code

Policy information about availability of computer code

Data collection	The fMRI data were acquired on a 3.0 T GE Signa scanner using a 32-channel head coil.
Data analysis	fMRI data were preprocessed using SPM12. Statistical analyses were performed using FSL6 and Matlab 2020.

For manuscripts utilizing custom algorithms or software that are central to the research but not yet described in published literature, software must be made available to editors and reviewers. We strongly encourage code deposition in a community repository (e.g. GitHub). See the Nature Portfolio guidelines for submitting code & software for further information.

Data

Policy information about availability of data

All manuscripts must include a data availability statement. This statement should provide the following information, where applicable:

- Accession codes, unique identifiers, or web links for publicly available datasets
- A description of any restrictions on data availability
- For clinical datasets or third party data, please ensure that the statement adheres to our policy

Original data reported in this study is available at https://openneuro.org/datasets/ds005899.

Research involving human participants, their data, or biological material

Policy information about studies with human participants or human data. See also policy information about sex, gender (identity/presentation), and sexual orientation and race, ethnicity and racism.

Reporting on sex and gender	Gender information was collected on self-report, and gender was taken as a covariate in this study.
Reporting on race, ethnicity, or other socially relevant groupings	We do not have access to race, ethnicity, or other socially relevant groupings information in the study.
Population characteristics	Age was included as a covariate in the data analysis.
Recruitment	Participants were recruited from a broad geographic region surrounding the San Francisco Bay Area through flyers.
Ethics oversight	Institutional Review Board of Stanford University

Note that full information on the approval of the study protocol must also be provided in the manuscript.

Field-specific reporting

Please select the one below that is the best fit for your research. If you are not sure, read the appropriate sections before making your selection.

[x] Life sciences [ ] Behavioural & social sciences [ ] Ecological, evolutionary & environmental sciences

For a reference copy of the document with all sections, see nature.com/documents/nr-reporting-summary-flat.pdf

Life sciences study design

All studies must disclose on these points even when the disclosure is negative.

Sample size	The sample size is determined based on samples from previous neuroimaging studies in children with and without ADHD and the effect is size is computed based on brain-behavior correlation analysis from previous studies using stop-signal tasks.
Data exclusions	Participants were excluded in the analysis if mean frame displacement were greater than 0.5mm and/or maximum displacement exceeded 5mm in either run. Participants with less than 75% accuracy on Go trials, or with greater than 75% or less than 25% accuracy on the Stop trials, or with longer RT in unsuccessful stop trials than go trials were excluded from further analysis to ensure accurate estimation of the SSRT.
Replication	The role of frontoparietal network and salience network in cognitive control were replicated by whole-brain searchlight and ROI-based analyses.
Randomization	The experimental groups are children with and without ADHD. There is no randomization issue.
Blinding	Blinding is not feasible because the experimental groups, children with and without ADHD, must be identified prior to their participation in the study.

Reporting for specific materials, systems and methods

We require information from authors about some types of materials, experimental systems and methods used in many studies. Here, indicate whether each material, system or method listed is relevant to your study. If you are not sure if a list item applies to your research, read the appropriate section before selecting a response.

Materials & experimental systems

n/a	Involved in the study	
[x]	[ ]	Antibodies
[x]	[ ]	Eukaryotic cell lines
[x]	[ ]	Palaeontology and archaeology
[x]	[ ]	Animals and other organisms
[x]	[ ]	Clinical data
[x]	[ ]	Dual use research of concern
[x]	[ ]	Plants

Methods

n/a	Involved in the study	
[x]	[ ]	ChIP-seq
[x]	[ ]	Flow cytometry
[ ]	[x]	MRI-based neuroimaging

Plants

Seed stocks	*Report on the source of all seed stocks or other plant material used. If applicable, state the seed stock centre and catalogue number. If plant specimens were collected from the field, describe the collection location, date and sampling procedures.*
Novel plant genotypes	*Describe the methods by which all novel plant genotypes were produced. This includes those generated by transgenic approaches, gene editing, chemical/radiation-based mutagenesis and hybridization. For transgenic lines, describe the transformation method, the number of independent lines analyzed and the generation upon which experiments were performed. For gene-edited lines, describe the editor used, the endogenous sequence targeted for editing, the targeting guide RNA sequence (if applicable) and how the editor was applied.*
Authentication	*Describe any authentication procedures for each seed stock used or novel genotype generated. Describe any experiments used to assess the effect of a mutation and, where applicable, how potential secondary effects (e.g. second site T-DNA insertions, mosiacism, off-target gene editing) were examined.*

Magnetic resonance imaging

Experimental design

Design type	event-related design
Design specifications	Each participant completes two runs of the cued stop-signal task and each run includes 80 trials.
Behavioral performance measures	Button press and response time was recorded in the experiment. The RACE mode was used to compute stop-signal reaction time (SSRT).

Acquisition

Imaging type(s)	functional
Field strength	3T
Sequence & imaging parameters	multiband gradient-echo planar imaging with the following parameters: TR=490ms; TE=30ms; flip angle=45°, FOV=22.2cm, matrix=74x74 and in-plane resolution=3mm.
Area of acquisition	32-channel head coil

Diffusion MRI ☐ Used ☒ Not used

Preprocessing

Preprocessing software	SPM12
Normalization	non-linear normalization was applied on nifti format data
Normalization template	MNI152 2mm template was used.
Noise and artifact removal	head motion was regressed out.
Volume censoring	visual inspection

Statistical modeling & inference

Model type and settings	representational similarity analysis; Spearman's correlation was used for examining brain-behavior relationship.
Effect(s) tested	Successful Stop versus Uncertain Go trials and Uncertain Go versus Certain Go trials are the main interests in the current study.

Specify type of analysis: ☐ Whole brain ☐ ROI-based ☒ Both

Anatomical location(s): ROIs were selected using coordinates from previous meta-analysis of inhibitory control.

Statistic type for inference (See Eklund et al. 2016)	FSL randomise, p<0.05 TFCE corrected.
Correction	FDR corrected.

Models & analysis

n/a	Involved in the study
☒	☐ Functional and/or effective connectivity
☒	☐ Graph analysis
☒	☐ Multivariate modeling or predictive analysis
